# Identification of Precise Therapeutic Targets and Characteristic Prognostic Genes Based on Immune Gene Characteristics in Uveal Melanoma

**DOI:** 10.3389/fcell.2021.666462

**Published:** 2021-05-26

**Authors:** Zhenxi Zhang, Jingyu Su, Li Li, Wenjing Du

**Affiliations:** State Key Laboratory of Medical Molecular Biology, Department of Cell Biology, Institute of Basic Medical Sciences, Chinese Academy of Medical Sciences, School of Basic Medicine, Peking Union Medical College, Beijing, China

**Keywords:** uveal melanoma, tumor microenvironment, immune classification, prognosis, precision therapy, PTGS2

## Abstract

The tumor microenvironment is an important factor for the immunotherapy of tumor patients. The sequenced transcriptome data can be used to describe the tumor microenvironment and various immune subtypes. We exploited published data on patients with uveal melanoma (UVM) to identify immune subtypes. Based on the immune-related gene sets of 80 patients with UVM in the TCGA database, we used consensus clustering to identify two immune subgroups. In the two immune subtypes, we analyzed clinical characteristics and immune infiltration. Class1 has low immune infiltration, contains memory B cells, Th2 cells, Th17 cells, eosinophils, natural killer cells, and has a better prognosis. Class2 has higher immune infiltration. CD8+ T cells, Th1 cells, MDSCs, and Dendritic cells are enriched in class2, which has strong cytolytic activity, high expression of immune checkpoint genes, and poor outcome. Moreover, we have developed and verified an immune characteristic model that can predict the prognosis of patients well. Through this model, we screened prostaglandin-endoperoxide synthase 2 (*PTGS2*) as the therapeutic target of UVM. Treatment of choroidal melanoma cell line (OCM1) cells with celecoxib (an inhibitor of *PTGS2*) effectively inhibits cell growth, proliferation, and promotes apoptosis. Our results show the immunological heterogeneity of UVM patients and also provide an ideal therapeutic target for the future treatment design of patients.

## Introduction

Uveal melanoma (UVM) is one of the most common primary intraocular malignant tumors, originating in the choroid, ciliary body, or iris (Willson et al., 2001; Chattopadhyay et al., 2016; Kaliki and Shields, 2017). It is estimated that there are about 7000 new cases of UVM every year worldwide, with an incidence rate of about 4.3 parts per million (Schank and Hassel, 2019). The 5-year survival rate of patients is 50–70%. However, UVM is very prone to metastasis. About 50% of patients will suffer from metastatic disease, which occurs more in the livers, lungs, and resulting in higher mortality (Willson et al., 2001; Kaliki and Shields, 2017). Although the treatment of tumor has been improved continuously, there is still no standard treatment method for metastatic UVM.

Recently, clinical trials have shown that immunotherapy is a promising therapy for many kinds of malignant tumors, including immune checkpoint blockade, cell therapy, and cytokine therapy (Yang, 2015; Callahan et al., 2016; Castellanos and Horn, 2016). Among them, immune checkpoint inhibitor therapy has been applied to treat a broad range of cancer types, such as lung cancer, liver cancer, and skin melanoma (Snyder et al., 2014; Johnson et al., 2017; Sugie, 2018). In recent years, some immunotherapies have also begun to treat UVM, such as ipilimumab, pembrolizumab and nivolumab, and adoptive cell therapy, alone or in combination (Ji et al., 2012; Karivedu et al., 2019; Jansen et al., 2020; Pelster et al., 2020). Unfortunately, the treatment effect for UVM was not ideal. In an immunotherapy study of 96 patients with uveal melanin, nivolumab and pembrolizumab alone or in combination therapy were used to treat 32, 54, and 15 patients, respectively. Only two patients had a partial response (PR) on nivolumab and combination therapy, and only one patient had a partial response (PR) on pembrolizumab. None of the three treatments had a complete response (CR) (Heppt et al., 2017). Chandran et al. (2017) found that only 30% of patients achieved a partial response in adoptive cell therapy for UVM (Chandran et al., 2017). Taken together, these results indicate that UVM is resistant to immunotherapy, and it is necessary to further explore the immunological mechanism in this cancer.

To better understand the tumor immune microenvironment, in this study, we divided UVM into two immune subgroups, based on consensus clustering of immune gene sets. The two immune subtypes had unique molecules, immune cell characteristics, and clinical outcome. We used Cox proportional hazard regression model to predict the survival of patients, and screened prostaglandin-endoperoxide synthase 2 *(PTGS2*) gene as a therapeutic target for UVM. Identifying the immune subtypes of UVM may contribute to a precision treatment for immunotherapy.

## Methods

### Patients and Datasets

The data for this study came from two databases (Table 1): The Cancer Genome Atlas (TCGA) and Gene Expression Omnibus (GEO). 80 UVM patients came from the TCGA database, containing RNA-seq data, somatic mutations, copy number variations (CNVs), DNA methylation and clinical sample data. 28 UVM patients came from GSE84976 containing RNA-seq data and clinical sample data (van Essen et al., 2016). 63 UVM patients came from GSE22138 containing RNA-seq data and clinical sample data (Laurent et al., 2011).

**TABLE 1:** Clinical characteristics of patients in the study.

**Characteristic**		**TCGA (*n* = 80)**	**GSE84976 (*n* = 28)**	**GSE22138 (*n* = 63)**
Age	≤50	18	7	10
	>50	62	21	53
Sex	male	45	N/A	39
	female	35	N/A	24
Stage	IIA	4	N/A	N/A
	IIB	32	N/A	N/A
	IIIA	27	N/A	N/A
	IIIB	10	N/A	N/A
	IIIC	3	N/A	N/A
	IV	4	N/A	N/A

### Identification of Immune Subtypes

Based on the previously reported expression of 782 immune-related genes, we used consensus clustering to identify the immune subtypes of UVM patients. The R package “ConsensusClusterPlus” was used for clustering. The following were some parameters, 80% items resampling, an evaluated K of 6, 50 resampling and others were the default values (Wilkerson and Hayes, 2010; Li et al., 2017). Cumulative distribution function (CDF) and consensus matrices were used to confirm the optimal number of subtypes.

### The Immune Characteristics Comparison of Two Immune Subtypes

Estimation of stromal and immune cells in malignant tumor tissues using expression data (ESTIMATE) was carried out to estimate the level of immune cell infiltration and somatic tumor score (Yoshihara et al., 2013). When using ESTIMATE, the sample file needed to be converted into a GCT format file as an input file, and GeneSymbol was used as the basis for gene identification. We used immune genes to establish gene sets of immune cells, cytolytic activity, TIL, and immune checkpoints. Immune cell score and immune characteristic score were quantified by the single-sample gene set enrichment analysis (ssGSEA) using R package “GSVA” (Hanzelmann et al., 2013).

### Differential Gene Analysis, Cox Proportional Hazard Regression Model

First, we used LIMMA to identify differential genes in count data of the two immune subtypes (Ritchie et al., 2015). The false discovery rate (FDR) < 0.05 was considered to be a differentially expressed gene. The R package “clusterProfiler” was used to analyze Gene Ontology (GO) (Yu et al., 2012).

A univariate Cox proportional hazard regression model was used to screen immune genes with prognostic value. We used the least absolute shrinkage and selection operator (LASSO) method for variable selection in the Cox regression model to determine meaningful prognostic genes and coefficient values. Using the linear combination of gene expression weighted regression coefficients, we got the risk score formula: risk score = (exp *PTGS2*^∗^3.88) + (exp *CCL24*^∗^0.32) + (exp *EPX*^∗^−1.25) + (exp *PAEP*^∗^0.03) + (exp *LY9*^∗^1.07) + (exp *PLXNB1*^∗^−0.007) + (exp *BDKRB2*^∗^0.4) + (exp *NKIRAS1*^∗^−0.19) + (exp *HLA-A*^∗^0.0003). The UVM patients were divided into low and high risk-score groups, and then the Kaplan–Meier method was used to analyze the relationship between risk scores and survival.

### Cell Culture and Cell Activity Test

Choroidal melanoma cell line OCM1 was obtained from the American Type Culture Collection (ATCC, United States). Cells were cultured in Dulbecco’s modified Eagle’s medium (DMEM) supplemented with 10% FBS, 100 U/mL penicillin, and 100 mg/mL streptomycin, at 37°C and 5% CO2. For cell proliferation assay, cells were seeded in 6-well cell culture dishes at a density of 10,000 cells/well, and treated with celecoxib (Solarbio, China). Cells were collected and counted at a fixed time every day for 6 days. For cell viability assays, cells were seeded in 96-well cell culture dishes at a density of 1,000 cells/well, and treated with celecoxib. One day later, CCK8 (Pplygen, China) was added into culture dishes for 3 h. OD_450__nm_ was measured using FlexStation 3. For cell clone experiment, cells were seeded in 6-well cell culture dishes at a density of 1,000 cells/well, and treated with celecoxib. After 10 days of culture, the cells were fixed with methanol for 30 min and stained with 0.1% crystal violet for 15 min. To detect apoptosis, cell culture media and cells were collected after 3 days. Apoptosis was detected by apoptosis kit (Keygentec, China).

### Statistical Analysis

All calculations and statistics were done using R. Unpaired *t*-test was used to detect two sets of samples with normal distribution. The Kaplan–Meier method was used for survival analysis. *P* < 0.05 was considered statistically significant.

## Results

### Immune Subtype Classification and Immune Cell Characteristics in UVM

**FIGURE 1:**
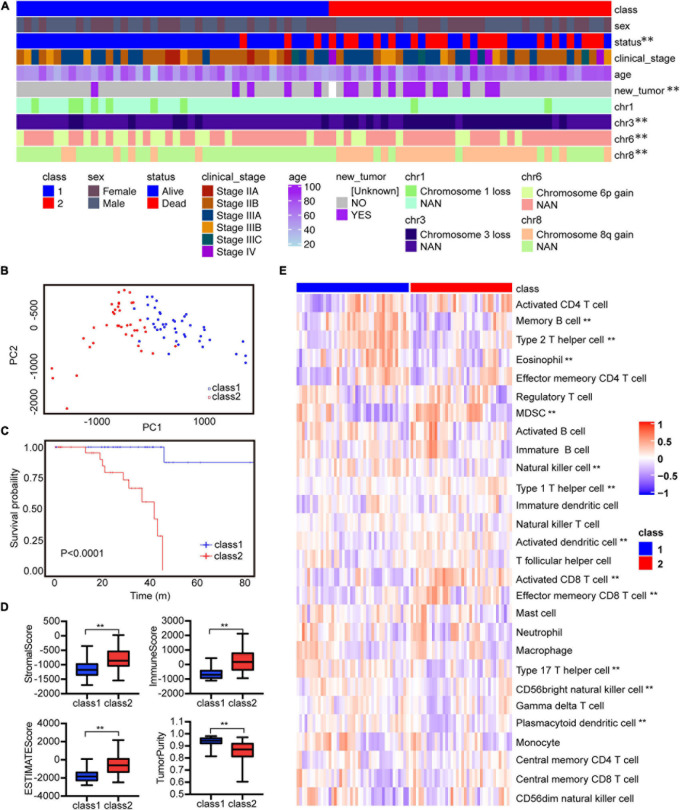
The two immune subtypes show different clinical characteristics outcomes and tumor immune infiltrating characteristics in TCGA. **(A)** Clinical characteristics of two immune subtypes. **(B)** PCA of two immune subtypes using 782 genes in TCGA. **(C)** Kaplan–Meier analysis of two immune subtypes based on overall survival. **(D)** Comparison of stromal, immune, ESTIMATE scores, and tumor purity scores for different immune subtypes in TCGA using ESTIMATE R package. **(E)** Heatmap of GSVA signature scores in TCGA. **P < 0.05.

In order to analyze the tumor microenvironment profile of UVM, we established an experimental diagram (Supplementary Figure 1A). Based on the consensus clustering analysis, TCGA UVM patients were divided into two immune types through immune gene expression profiles (Supplementary Figures 1A,B). Principal component analysis (PCA) was applied to further verify the difference in gene expression between these two immune types (Figure 1B). In these two immune subtypes, there were significant differences among the survival status of patients. Class2 patients had more deaths, higher rate of stage III and IV, higher risk of new tumor growth, in addition chromosome 3 loss and chromosome 8q gain (Figures 1A,C). While Class1 was associated with better survival rate (Figure 1C) and enriched in chromosome 6p gain (Figure 1A).

To analyze the immune characteristics of the two immune subtypes, we adopted two immune-related tools for analysis. We used the ESTIMATE method to compare the immune scores of these two subtypes. Class2 had higher stromal, immune and estimate scores but lower somatic cell purity (Figure 1D). Further, we applied the ssGSEA method to analyze the enrichment level of immune cells in immune subtypes. Class1 had higher amounts of memory B cells, Th2 cells, Eosinophils, natural killer (NK) cells, Th17 cells, CD56 bright natural killer cells, and Plasmacytoid dendritic cells (Figure 1E). Class2 was enriched in MDSCs, TH1 cells, activated dendritic cells, activated CD8 T cells and effector memory CD8 T cells (Figure 1E). Generally, human leukocyte antigen (HLA) and major histocompatibility complex (MHC) molecules activate CD8 T cells during antigen-specific immune responses (Garrido and Aptsiauri, 2019). Therefore, we further analyzed the changes of immune characteristics between these two subtypes. Higher expression levels of HLA and MHC molecules, more cytotoxicity, Tumor infiltrating lymphocytes (TIL) infiltration, and T cell costimulation were found in class 2 (Supplementary Figure 2A). Whereas in class1 there was a higher level of activated TNF-II molecule. In addition, immune checkpoint suppression therapy has become an effective tumor treatment method, so we compared the immune checkpoint gene expression between these two subgroups (Parry et al., 2005; Taggart et al., 2018). Tumor immune checkpoint genes such as *LAG3*, *IDO1*, *PDCD1* were highly expressed in class2 (Supplementary Figure 2B). Above all, these results indicate that class1 has a moderate immune microenvironment, while class2 has a high immune infiltration and the degree of immunosuppression.

### Prognostic Associations of Tumor Immune Characteristics

**FIGURE 2:**
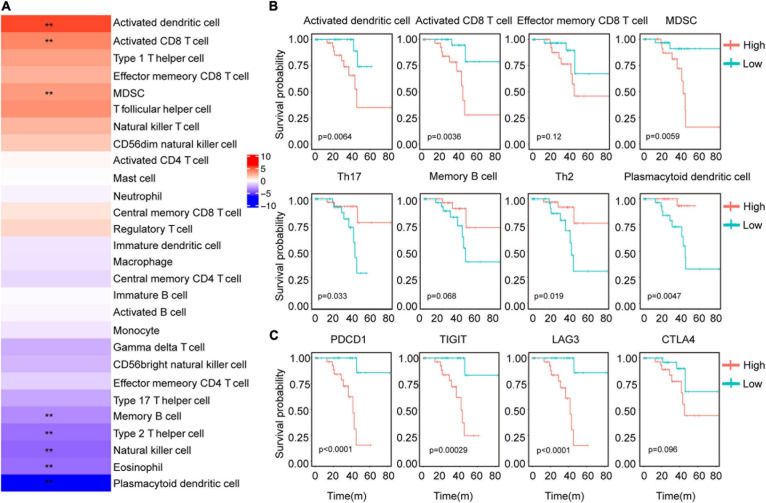
Correlation between immune characteristics and clinical outcomes of UVM. **(A)** Heatmap showed the risk ratio of the immune cell characteristic score in the immune subtypes. **(B)** Kaplan–Meier analysis of the correlation between the scores of activated CD8 T cells, Th1 cells, Th2 cells, Th17 cells, NK cells, activated B cells, and OS. **(C)** Kaplan–Meier analysis of the correlation between gene expression of *LAG3*, *PDCD1*, *TIGIT*, and OS. The expression above the median is high and below the median is low. **P < 0.05.

Tumor infiltrating immune cells play an important role in many tumors for immunotherapy. Next, we detected the effect of immune cells in the prognosis of UVM. Among these immune characteristics, memory B cells, Th2 cells, NK cells, eosinophils and plasmacytoid dendritic cells, activated dendritic cells, activated CD8 T cells and MDSCs were related to prognosis (Figure 2A). Consistently with the Cox analysis, more activated dendritic cells, activated CD8 T cells and MDSCs were associated with a poor prognosis. High levels of Th17 cells, Th2 cells, and plasmacytoid dendritic cells linked with good prognosis (Figure 2B). In addition, we evaluated the prognosis of major immune checkpoints and found that high expression of *LAG3*, *PDCD1*, and *TIGIT* led to worse outcomes (Figure 2C). We further analyzed the correlation between immune cells and immune checkpoint genes. The data showed that immune checkpoint genes were positively correlated with these immune cells with poor prognosis, and negatively correlated with these immune cells with good prognosis (Supplementary Figure 3A). We analyzed the correlation of 28 immune cells. Activated CD8 T cells were positively correlated with effector memory CD8 T cells, MDSC cells, and negatively correlated with Plasmacytoid dendritic cells (Supplementary Figure 3B). These results suggest that infiltrating immune cells could affect the patient’s outcome, and probably provide an idea for targeted immunotherapy of UVM.

### Development and Validation of an Immune-Related Risk Model Using Cox Proportional Hazards Model

**FIGURE 3:**
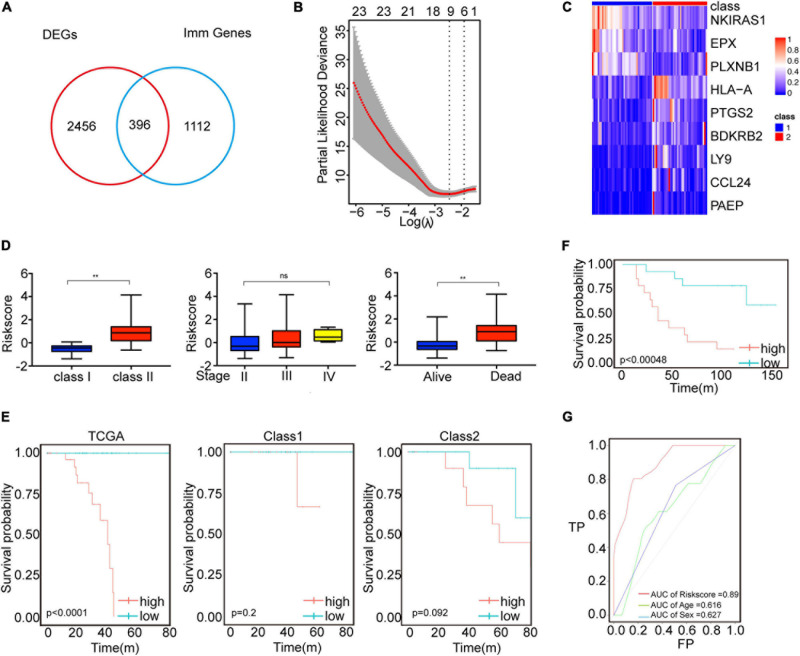
Immune characteristics were identified by Cox proportional hazard model. **(A)** Venn diagram showed immune-related genes which are differentially expressed between the two immune subtypes. **(B)** Cross-validation for parameter selection in adjusted proportional hazard models. **(C)** Heatmap showed the expression level of characteristic genes. **(D)** Distribution of risk scores for immune subtypes, tumor stage, patient status. **P < 0.05. **(E)** Survival analysis of risk scores in UVM immune subtypes. **(F)** Survival analysis of the risk score of GSE84976. **(G)** ROC curve analysis of age, sex, and immune score.

We next validated the survival model using the Cox proportional hazard model. We identified 2852 differentially expressed genes between class1 and class2 subtypes, of which 396 were immune genes by LIMMA (Figure 3A). We did GO analysis using differential genes. In Class2, the up-regulated genes were mainly enriched in receptor ligand activity, CCR chemokine receptor binding and MHC protein complex binding pathways (Supplementary Figure 1E). Using univariate Cox regression analysis, it was found that 306 genes were significantly related to the patient’s prognosis. The best prognostic value model related to Cox was established via GLMNET (Figure 3B). The model obtained nine immune gene signals (Figure 3C), and we used this model to calculate the score of UVM patients. Compared to class1, class2 had a higher risk score (Figure 3D). In addition, we found that the risk score of UVM in the stage IV was higher than stage II (Figure 3D). Kaplan–Meier analysis showed that patients with high-risk risk scores had worse survival (Figure 3E). Similar result was obtained using GEO database data GSE84976 and GSE22138 (Figure 3F and Supplementary Figure 4A). However, in order to verify the adaptability of this prediction model, we verified three TCGA databases, Brain Lower Grade Glioma (TCGA-LGG), Skin Cutaneous Melanoma (TCGA-SKCM), and Liver Hepatocellular Carcinoma (TCGA-LIHC). This model can’t effectively predict tumor models other than UVM (Supplementary Figures 4B–D). The area under the curve (AUC) validated that the immune risk model had a better predictive effect (AUC = 0.89) than age and sex predictions (Figure 3G). These results suggest that the immune gene prediction model has a good predictive effect in prognostic prediction.

### PTGS2 Is a Potential Target for the Uveal Melanoma Therapy

**FIGURE 4:**
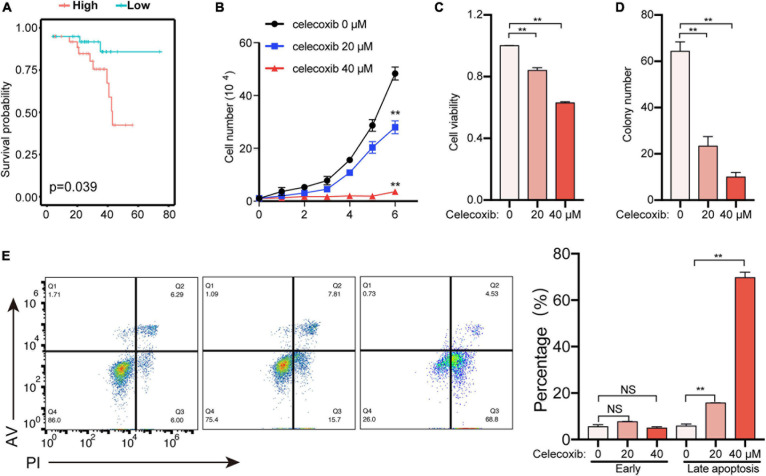
PTGS2 is a potential therapeutic target to treat uveal melanoma. **(A)** Survival analysis of *PTGS2* expression in UVM. The *PTGS2* expression above the median is defined as high and below the median is defined as low. **(B)** OCM1 cells growth curve was detected for 6 days after celecoxib treatment. **(C)** OCM1 cells viability was detected using CCK8 after celecoxib treatment for 1 day. **(D)** OCM1 cells clone formation was detected. The number of cell clones was more than 2 mm in diameter. **(E)** OCM1 cells apoptosis was detected after celecoxib treatment for 3 days. **P < 0.05.

In order to screen precision therapy targets for the treatment of uveal melanoma, we screened genes in the prediction model for verification. The previous risk prediction model showed that *PTGS2* (Cyclooxygenase-2, COX2) had the highest coefficient. Meanwhile, in UVM patients, high expression of *PTGS2* had a worse prognosis (Figure 4A). Therefore, *PTGS2* may be an effective therapeutic target. In order to explore the role of *PTGS2* in tumor cells, we used celecoxib, a selective inhibitor of *PTGS2*, to detect cell viability in OCM1 cells. Celecoxib effectively inhibited cell proliferation and cell viability in a dose-dependent way (Figures 4B,C). Colony formation experiment also showed celecoxib decreased cell growth (Figure 4D). Next, we examined the effect of celecoxib on cell apoptosis. As shown in Figure 4E, celecoxib treatment promoted late apoptosis dose-dependently. Taken together, these data indicate that *PTGS2* may be a potential therapeutic target for uveal melanoma treatment.

## Discussion

Clinical practice studies have shown that the effect of immunotherapy in the treatment of UVM is limited. A better understanding of the immune microenvironment of uveal tumors can help to improve the effectiveness of immunotherapy. Here we introduced the immunological features of UVM. Our results indicate that UVM can be divided into two immune subtypes (class1 and class2). Each immune subtype has unique immune cells and immune functions. Moreover, the clinical characteristics of these two immune subtypes are significantly different. We used the risk model to predict the survival of patients, and screened *PTGS2* gene as a therapeutic target for UVM.

Class1 had a good prognosis, with higher Eosinophils, NK cells, Th17 cells, and Plasmacytoid dendritic cells infiltration. In some studies, these cells are beneficial to tumor infiltrating lymphocytes for killing tumor. The increase in Th17 cells significantly improves patient survival (Asadzadeh et al., 2017; Qian et al., 2017). Eosinophils promote immune response and there are more NK cells in the tumor, which can kill the tumor better (Malmberg et al., 2017; Moreira and Heinzerling, 2017; Cozar et al., 2020). Class2 had a worse outcome, with higher lymphocytes, MDSCs infiltration, immune checkpoint genes expression. These results indicate that class2 has a tumor microenvironment with high immune infiltration and high immunosuppression (Kumar et al., 2016; Sun et al., 2018). Therefore, for the patients in this subgroup of class 2, we can use immune checkpoint therapy to effectively improve the prognosis (Callahan et al., 2016). Moreover, the existing immunotherapy has no effect on some patients, which may be because these patients belong to class1. However, the gene expression profile data for immunotherapy needs to be further verified.

Considering the inability of single factor to predict prognosis, we used an elastic-net regression Cox model to screen out significant immune genes and constructed a prognostic prediction model. Among the differential genes of immune subtypes, there were 306 immune-related genes that were related to survival. Nine genes were screened out from 306 genes and used to construct a prediction model. In TCGA’s UVM data, low risk-score and high risk-score show significant survival differences, and high risk-score patients have worse outcomes. This model has good predictive accuracy. At the same time, the applicability of the model was not only verified using GEO data, but also confirmed with TCGA data. Of course, this risk model needs more data and experiments to verify.

In order to screen therapeutic targets, we selected the key gene *PTGS2* from Cox model. *PTGS2* is expressed in many tumors and plays a role in tumorigenesis, tumor metastasis, and tumor treatment resistance (Ching et al., 2020). *PTGS2* plays a major role in promoting the proliferation, invasion, metastasis and anti-apoptosis of cancer cells through its metabolite prostaglandin E2. The PTGS2-PGE2-EP signaling pathway inhibits NK cells and T cells, and promotes tumor immune escape (Liu et al., 2015). In pancreatic cancer, knocking out PTGS2 or using its inhibitors can help the tumor to be sensitive to immunotherapy (Markosyan et al., 2019). *In vitro*, celecoxib treatment effectively inhibited OCM1 cells proliferation, cell viability, and promoted cell apoptosis. Whether Celecoxib treatment of OCM1 cells affects tumor immunotherapy remains to be verified. Celecoxib has been used for more than 20 years, mainly for the treatment of rheumatoid arthritis and other inflammatory diseases (Toloczko-Iwaniuk et al., 2019). In recent years, there are more and more researches on celecoxib for cancer treatment, such as breast cancer, colorectal cancer, and pancreatic cancer (Zuo et al., 2018; Toloczko-Iwaniuk et al., 2019). Although the clinical efficacy of celecoxib in the treatment of uveal melanoma needs further to be investigated, our data suggest celecoxib may be effective for treating uveal melanoma.

## Conclusion

In our study, UVM can be divided into two immune subtypes. The identification of immune subtypes in UVM establishes a risk prediction model, which effectively predicts the prognosis of patients. Moreover, celecoxib treatment can effectively inhibit the proliferation of OCM1 cells and promote cell apoptosis. Thus, *PTGS2* is a potential target of precision therapy for UVM. Our work is conducive to the understanding of the tumor immune microenvironment of UVM, and also provides valuable information for patients’ personalized immunotherapy.

## Data Availability Statement

All data supporting this study were openly available from TCGA database (http://cancergemome.nih.gov/) and GEO database (https://www.ncbi.nlm.nih.gov). The accession number(s) can be found in the article/Supplementary Material.

## Author Contributions

WD, LL, and ZZ conceived and designed the study. ZZ analyzed the data. LL, ZZ, and JS participated in the writing and revision of article. All authors contributed to the article and approved the submitted version.

## Conflict of Interest

The authors declare that the research was conducted in the absence of any commercial or financial relationships that could be construed as a potential conflict of interest.

## Supplementary Material

Supplementary Figure 1 Consensus clustering based on immune gene expression of 80 UVM in TCGA. **(A)** Flow chart of the study. **(B)** Clustering matrix for *K* = 2. **(C)** CDF curve for *k* = 2 to *k* = 6. **(D)** Delta area for *k* = 2 to *k* = 6. **(E)** GO analysis of genes upregulated in class2.

Supplementary Figure 2 **(A)** Heatmap of immune function score. **(B)** Heatmap of immune checkpoint related genes expression. ***P* < 0.05.

Supplementary Figure 3 **(A)** Heatmap of the correlation between immune cells and immune checkpoints. **(B)** Heatmap of the correlation between immune cells.

Supplementary Figure 4 **(A)** Survival analysis of the risk score of GSE22138. **(B)** Survival analysis of the risk score of TCGA-LGG. **(C)** Survival analysis of the risk score of TCGA-SKCM. **(D)** Survival analysis of the risk score of TCGA-LIHC.
