# Supplementary figures and images for: Identification of Precise Therapeutic Targets and Characteristic Prognostic Genes Based on Immune Gene Characteristics in Uveal Melanoma
Source: Front Cell Dev Biol. 2021 May 26;9:666462. doi: 10.3389/fcell.2021.666462 (PMC8187912; doi:10.3389/fcell.2021.666462)

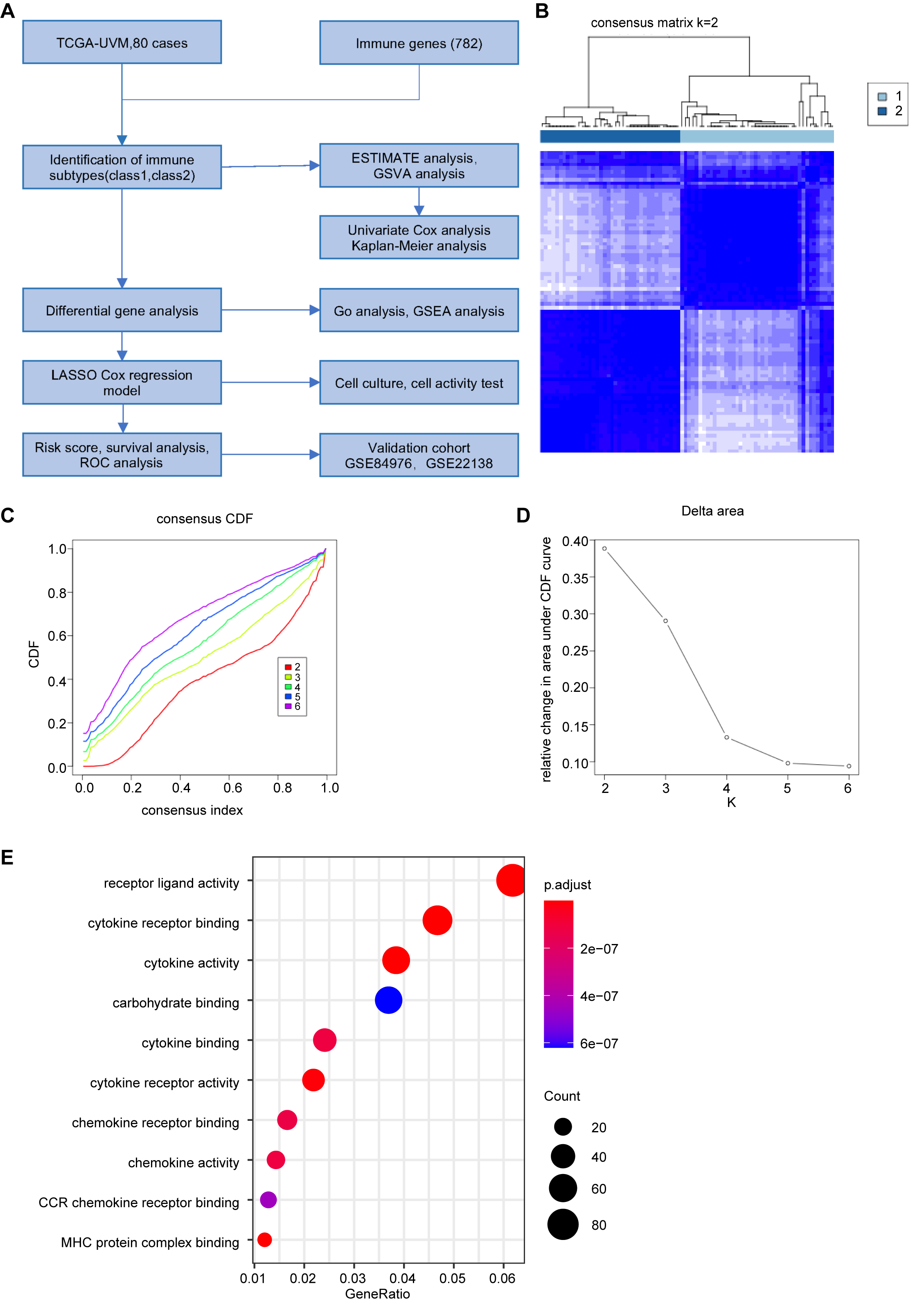

Supplement: Supplementary Figure 1 — Consensus clustering based on immune gene expression of 80 UVM in TCGA. (A) Flow chart of the study. (B) Clustering matrix for K = 2. (C) CDF curve for k = 2 to k = 6. (D) Delta area for k = 2 to k = 6. (E) GO analysis of genes upregulated in class2. [file Image_1.TIF]

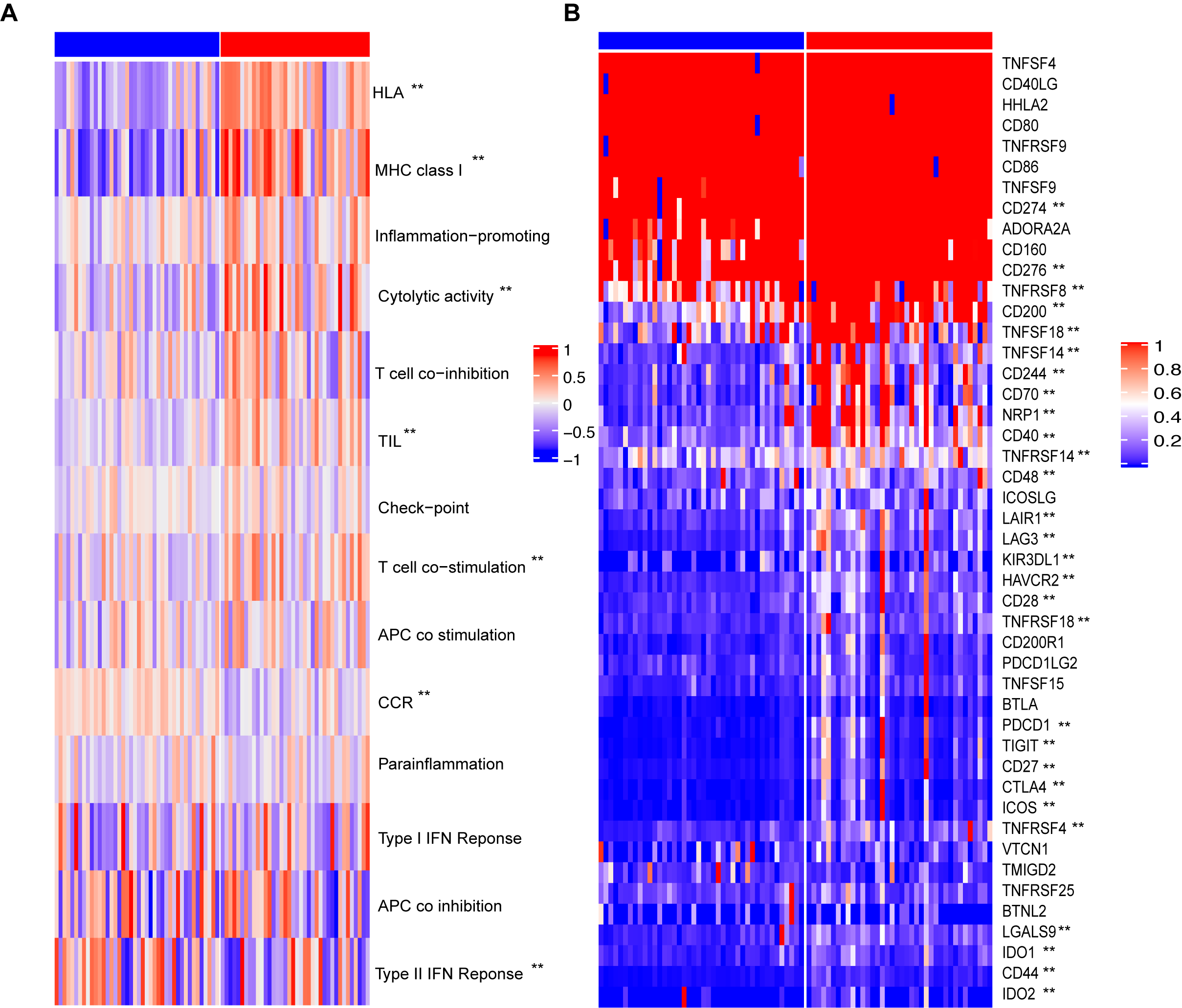

Supplement: Supplementary Figure 2 — (A) Heatmap of immune function score. (B) Heatmap of immune checkpoint related genes expression. **P < 0.05. [file Image_2.TIF]

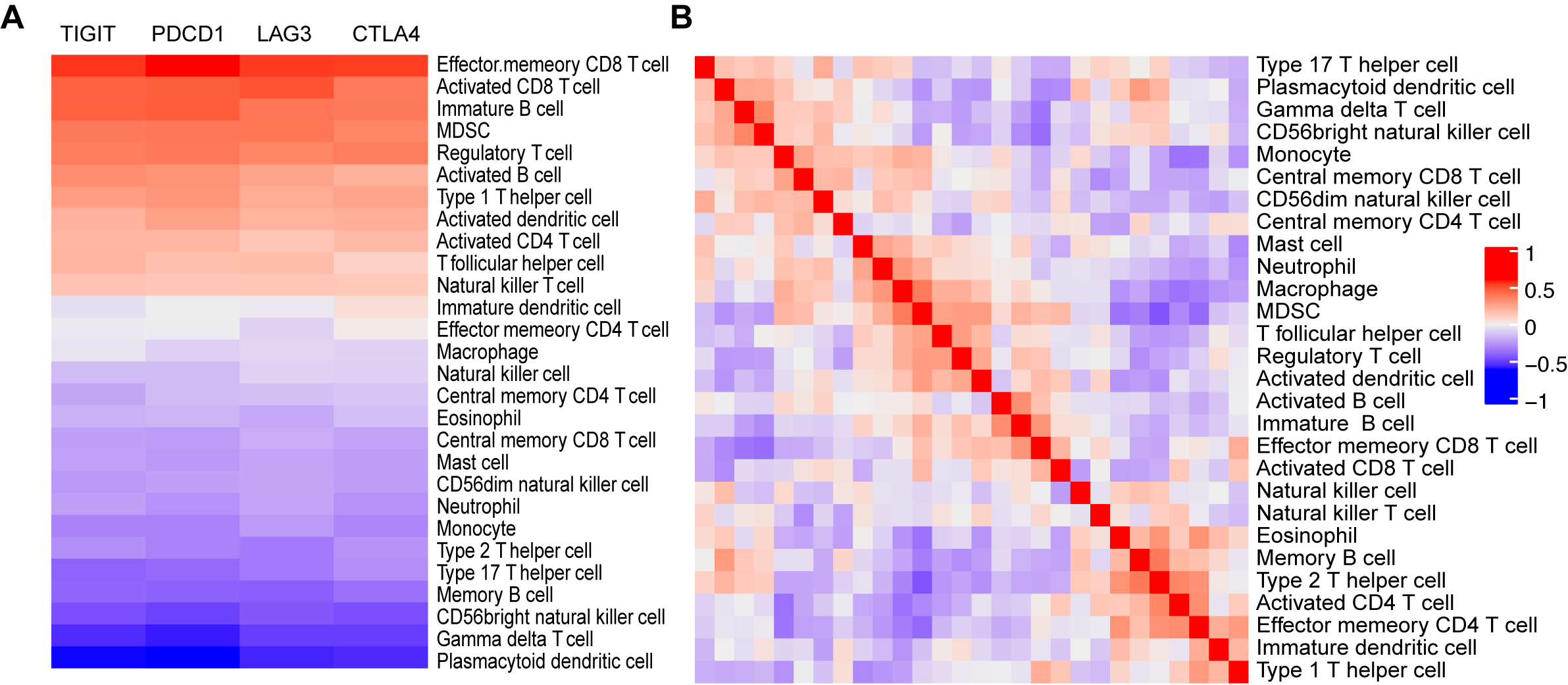

Supplement: Supplementary Figure 3 — (A) Heatmap of the correlation between immune cells and immune checkpoints. (B) Heatmap of the correlation between immune cells. [file Image_3.TIF]

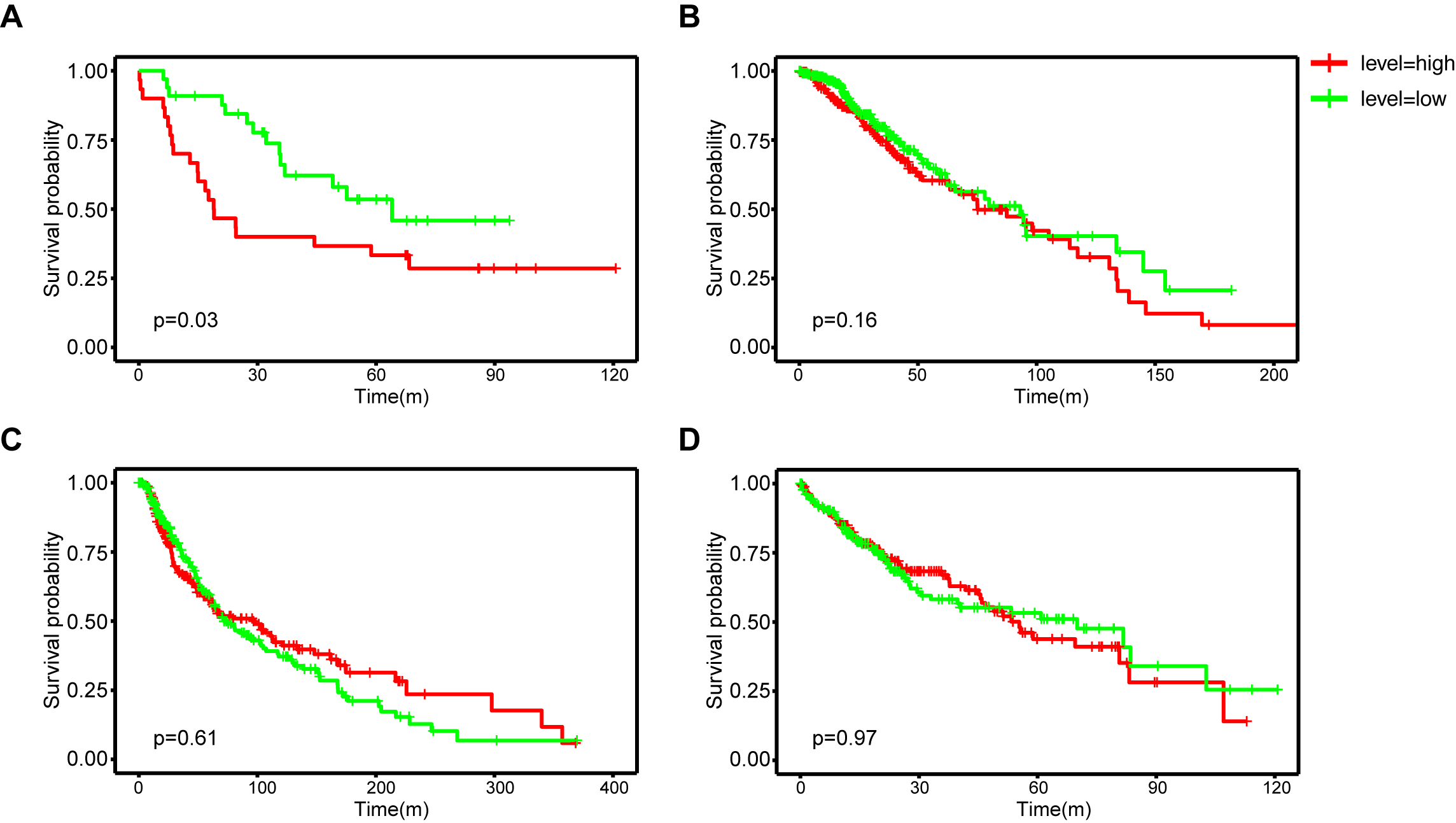

Supplement: Supplementary Figure 4 — (A) Survival analysis of the risk score of GSE22138. (B) Survival analysis of the risk score of TCGA-LGG. (C) Survival analysis of the risk score of TCGA-SKCM. (D) Survival analysis of the risk score of TCGA-LIHC. [file Image_4.TIF]
